# Nedaplatin reduces multidrug resistance of non-small cell lung cancer by downregulating the expression of long non-coding RNA MVIH

**DOI:** 10.7150/jca.35792

**Published:** 2020-01-01

**Authors:** Changwen Jing, Zhuo Wang, Rui Lou, Jianzhong Wu, Chen Shi, Dan Chen, Rong Ma, Siwen Liu, Haixia Cao, Jifeng Feng

**Affiliations:** 1 Research Center for Clinical Oncology, Jiangsu Cancer Hospital & Jiangsu Institute of Cancer Research & The Affiliated Cancer Hospital of Nanjing Medical University, Nanjing, Jiangsu Province, China; 2 Department of Oncology, Jiangsu Cancer Hospital & Jiangsu Institute of Cancer Research & The Affiliated Cancer Hospital of Nanjing Medical University, Nanjing, Jiangsu Province, China

**Keywords:** nedaplatin, lncRNA, MVIH, NSCLC, multidrug resistance, EMT

## Abstract

Cisplatin-based chemotherapy is the standard treatment for non-small cell lung cancer (NSCLC). However, drug resistance emergences after treatment. Long non-coding RNA microvascular invasion in hepatic cancer (MVIH) plays an important role in drug resistance in a variety of cancers. This study investigates the role of nedaplatin on multidrug resistance in NSCLC and its relationship with MVIH. Lung cancer A549 and H1650 cells were treated with cisplatin to obtain multidrug-resistant A549/DDP and H1650/ DDP cells. A549/DDP and H1650/ DDP cells were treated with nedaplatin, MVIH siRNA and siRNA NC. It was found that both MVIH siRNA and nedaplatin significantly reduce the mRNA expression of MVIH in A549/DDP and H1650/ DDP cells. MTT assay showed that the proliferation of MDR cells was significantly higher than that of other cells. Nedaplatin and MVIH siRNA significantly inhibit the proliferation of A549 and H1650 cells. The results of colony formation assay were consistence with MTT results. Nedaplatin and MVIH siRNA significantly reduced colony formation in MDR cells. Flow cytometry showed that NDP and MVIH siRNA significantly decrease the proportion of cells in G0/G1 and increase the proportion of cells in S phase compared with untreated and MDR cells. The apoptotic rate of MDR cells was significantly lower than that of other cells, while the apoptosis rate of cells in NDP and MVIH siRNA group was significantly higher than that of the other three groups of cells. Wound healing assay and Transwell chamber experiments confirmed that both NDP and MVIH siRNA significantly reduced the migration and invasion abilities of MDR cells. The expression of E-cadherin in MDR cells was significantly lower than that in untreated cells, and the expression of N-cad, α-SMA and Vimentin significantly increased in the MDR cells. NPD and MVIH siRNA reverse the EMT process. In conclusion, LncRNA MVIH is upregulated in drug resistant NSCLC cells. Nedaplatin can reduce the expression of MVIH and reverse EMT process, thus reversing the drug resistance of cisplatin in non-small cell lung cancer cells.

## Introduction

Non-small cell lung cancer (NSCLC) is the second most common cancer around the world in both men and women 1. Typical treatment for NSCLC includes surgical operation, radiation and chemotherapy. Cisplatin and cisplatin-based combination chemotherapy has become the standard treatment for NSCLC because of its superior efficacy, acceptable side effects and relatively low cost 2. However, due to the heterogeneity of lung cancer, some patients are not sensitive to cisplatin treatment or drug resistance could be acquired after a period of chemotherapy, resulting in treatment failure and tumor recurrence. Therefore, understanding the mechanism of acquisition of cisplatin resistance in NSCLC cells would benefit patient in clinical settings 3.

In recent years, studies have shown that patients with different genomic alteration or epigenetic profiles have distinct responses to therapeutics 4-6. Long non-coding RNA (lncRNA) plays an important role in gene expression and regulates tumor formation, proliferation, invasion and migration 7. Recent studies have found that lncRNA associated with microvascular invasion in hepatocellular carcinoma (MVIH) was abnormally expressed in a variety of cancer cells 8-10. In breast cancer, overexpressed MVIH is associated with poor survivals. Inhibition of MVIH reduced the hepatocellular carcinoma proliferation. However, whether MVIH is directly involved in the regulation of drug resistance has not yet been investigated.

Nedaplatin (NDP) is an analog of cisplatin (DDP), which is capable of binding to DNA and inhibiting DNA replication in the same manner as DDP. A phase II clinical study reported on NDP treatment for non-small cell lung cancer 11 suggested that NDP have a certain effect on some cisplatin or carboplatin-resistant non-small cell lung cancer patients, indicating that NDP, DDP and carboplatin do not have full cross-resistance. Currently, there are few studies on the biological function and effect of NDP on non-small cell lung cancer cells, and the mechanism of NDP' on tumor drug resistance is not available.

In this study, we found that lncRNA MVIH is a differentially expressed between non-chemotherapy treated and chemotherapy treated NSCLC cells. We investigated the function of MVIH in two NSCLC cell lines (A549 and H1650) to explore whether they have the capacity to mediate NSCLC chemotherapeutic resistance.

## Materials and Methods

### Cell culture

Non-small cell lung cancer cell lines A549 and H1650 were purchased from Shanghai Cell Bank of the Chinese Academy of Sciences and cultured in DMEM medium containing 10% FBS (GIBCO, USA). The culture environment was 5% CO_2_, at 37℃. Nedaplatin (NDP) was purchased from Nanjing Dongjie Pharmaceutical Co., Ltd., and 5mL of saline was used to prepare NDP with the concentration of 2mg/mL as stock solution. The samples were stored at a -20℃ refrigerator until use. Cisplatin resistant A549 (A549/DDP) and H1650 (H1650/DDP) cells were cultured in the same conditions as A549 cells and H1650 cells. To maintain the resistance to cisplatin, cells were cultured in medium containing 2μg/mL cisplatin. Cells in the logarithmic phase were collected for the following experiments.

### Cell grouping and transfection

Cells were grouped as follow: Blank (untreated A549 and H1650 cells); MDR (A549/DDP cells and H1650/DDP cells); NDP (Nedaplatin-treated A549/ DDP cells and H1650/DDP cells); MVIH siRNA (MVIH siRNA transfected A549/DDP cells and H1650/DDP cells); siRNA NC (siRNA vehicle transfected A549/DDP cells and H1650/DDP cells). Si-MVIH and Non-specific siRNA (si-NC) were purchased from Invitrogen (Carlsbad, CA, USA). All plasmid vectors (pCDNA-MVIH and empty vector pCDNA) were transfected into A549 and H1650 cells using Lipofectamine2000 (Invitrogen, Shanghai, China) in accordance with the instruction of manufacturers. Cells were harvested after 48 hours for qRT-PCR or Western blot analyses.

### Quantitative PCR

All PCR primers were designed with Primer 5.0 software and were synthesized by Shanghai GenePharma Company (Shanghai, China), according to the gene sequences in GenBank. A real-time PCR System (ABI Company, Oyster Bay, NY) and SYBR Green I fluorescence kit (Takara Biotechnology Ltd., Dalian, China) were used for PCR reaction. Standard curves were plotted for each PCR reaction to confirm the reliability. All expression of genes was normalized to *GAPDH* which is an internal control. Once the CT value (amplification power curve inflection point) was obtained, we calculate the gene expression using the following formula: △Ct = CT (target gene) - CT (internal reference), △△Ct = △Ct (treatment group) - △Ct (control group); the relative expression of target genes was calculated using 2^-△△^Ct. The forward and reverse primers of indicated genes are as follows:

### Western Blot analysis

Proteins were extracted from tissue samples, and a bicinchoninic acid (BCA) kit (Wuhan Boster Biological Technology Co. Ltd., Wuhan, China) was used to evaluate the protein concentration. After the sample buffer was added to 30 µg of the proteins, the proteins were boiled at 95°C for 10 min. Afterwards, the proteins were separated using electrophoresis. After electrophoresis, proteins were transferred onto polyvinylidene fluoride (PVDF) membranes with 100 V transfer-molded voltage lasting for 45 to 70 min. Once the proteins were transferred into the membranes, membranes were incubated at room temperature for 1 h with 5% bovine serum albumin (BSA) for blocking. Membranes were incubated with primary antibodies (1: 1000 dilution) (purchased from Abcam Inc., Cambridge, MA, USA), on a shaker overnight at 4°C. Then, samples were washed with tris-buffered saline Tween 20 (TBST) 3 times (5 min/time). The corresponding secondary antibody was added for incubation at room temperature for 1 h, then membranes were washed 3 times. After that, membranes were subjected to a developer for imaging using chemiluminescence reagents. GAPDH and beta-actin were used as internal references. Bands were visualized with a Bio-Rad Gel Doc EZ imager (GEL DOC EZ IMAGER, Bio-Rad, California, USA). Image J software was used to analyze the intensity of the target bands.

### MTT assay

Cells in the logarithmic growth phase were collected and seeded into a 96-well plate with the cell concentration of 5 × 10^3^ cells/mL. After the cells had adhered to the bottom of the plate, they were cultured for 24, 48 and 72 h, and then 10 μL MTT was applied into the cell culture media. After another 4 h incubation, the culture solution was discarded. Subsequently, a total of 150 μL dimethyl sulfoxide (DMSO) was added, and the plates were shaken for 10 min in the dark. Then, the light absorbance value at a wavelength of 450 nm was detected.

### Cell colony formation assay

RPMI 1640 medium supplemented with 10% FBS and 0.6% agar was added into the 6-well plate 12. Plates were kept at room temperature for 10 min. After solidification, a total of 1×10^3^ cells/mL cells were seeded with RPMI 1640 medium supplemented with 10% FBS and 0.3% agar. After 14 days of culturing at 37°C, colony formation was observed. Cell numbers ≥50 were counted as a colony. The numbers of colonies were counted by randomly selecting 5 fields in each group. Colony forming efficiency (CFE %) was defined as the ratio of the number of colonies formed in culture to the number of cells inoculated.

### Apoptosis analysis

To examine the proportion of cells in each phase of cell cycle, cells were suspended and fixed on ice for 15 min with 1 mL of cold 70% ethanol after trypsinization and washed by PBS. The cells were subsequently centrifuged and the cell pellets were re-suspended in 1 mL of propidium iodide (PI) solution (0.05 mg/mL PI, 0.02 mg/mL RNase, 0.3% NP40 1mg/mL sodium citrate) for 1 h at 4℃. Flow cytometry analysis was performed using a FAC Sort flow cytometer.

### Wound healing assay

Matrigel diluent was paved in 24-well plate, then the plates were placed at room temperature with air drying for 30 min. Cells were inoculated in the prepared plate and cultured in an incubator (37℃, 5%CO_2_). When cells fully covered the bottom of plates, a line was lightly drawn in each well with a sterilized 200 μL tip, ensuring the width of each line was identical. A mark was left on the cap of the 24-well plates to make sure the same visual field in the photograph, and then typical images were taken and recorded as 0 h. After 24 h incubation, the 24-well plate was washed 3 times with PBS to remove any cell debris. Afterwards, cells were put into a serum-free medium for photograph, and recorded as 24 h. Photographs were taken with Olympus Inverted Microscope (Olympus Optical Co., Ltd, Tokyo, Japan) with 6 visual fields at a fixed location. The migrated distance was calculated with the Image Tool software (Bechtel Nevada, Los Alamos Operations, USA). Migrated distance= (width at 0 h - width at 24 h)/2.

### Transwell cell invasion assay

Each Transwell chamber (Corning Glass Works, Corning, New York, USA) was added with matrigel (3.9 mg/ml, 60~80 μl), and incubated at 37°C. When the matrigel was solidified, chambers were taken out and put into a 24-well plate. The pre-warmed mediums (0.5 mL per chamber) were added into upper and lower chambers separately. Then the chambers were put into the incubator for another 2 h, followed by the media removed from upper and lower chambers. The cell suspension (5 × 10^4^ cells/mL) was prepared after digestion. A total of 0.5 mL complete medium was extracted and put into a 24-well plate, and then the chamber was transferred into the 24-well plate. A total of 0.5 mL cell suspension was extracted and put into the chamber, followed by incubation at 37°C for 24 h. After 24 h culturing, the liquid in the upper and lower chambers was discarded. Cotton swabs were used to clean the cells on the surface of the upper chamber of the Transwell membrane. After three times of PBS washing, the transferred cells were fixed with cold methanol for 30 min. Cells were then dyed with 0.1% crystal violet for 10 min. Subsequently, cells were washed by tap water until no extra crystal violet remained. Finally, data were recorded through a microscope. Photographs were captured using Olympus Inverted Microscope (Olympus Optical Co., Ltd., Tokyo, Japan) with 6 visual fields at a fixed location. A hit counter was applied to count the number of cells transferred onto the lower chamber.

### Animal experiments

All mouse experiments were performed in accordance with animal ethics approved by our hospital's ethics committee. A total of 30 SCID mice were randomly assigned to 5 groups (n = 6 per group). Cells were pre-treated with cisplatin (MDR), nedaplatin (NDP), MVIH siRNA (MVIH siRNA) or siRNA controls (siRNA NC) before implanted into mice. A 27-gauge needle was applied to subcutaneously inject 500,000 A549 cells (siRNA treated or platin treated or untreated) into each mouse. Tumor size was measured every 3 days after one week post implantation. Mice were scarified when tumors reached a volume of maximal 5000 mm^3^.

### Statistical analysis

All data were analyzed by GraphPad Prism version 6 statistical software. Measurement data were expressed by mean ± standard deviation. The t test was used for comparisons between two groups. One- way analysis of variance was applied for comparisons of multiple groups. Statistical significance was set at *P* < 0.05.

## Results

### LncRNA MVIH siRNA and NDP treatment significantly decreased lung cancer cell proliferation and colony formation

**Figure 1:**
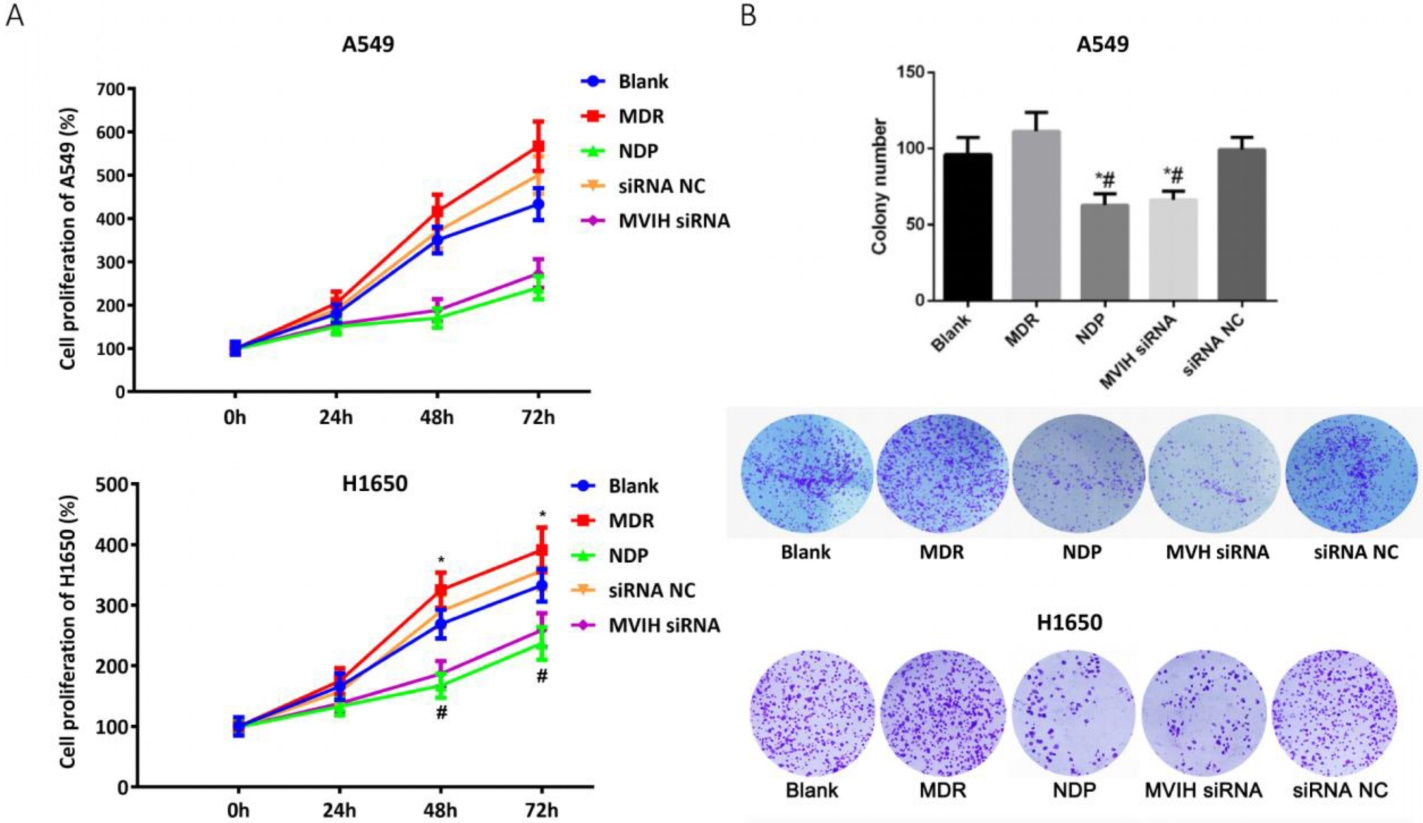
** Cell proliferation after nedaplatin treatment and knockdown of MVIH.** A) MTT assay. B) Colony formation assay. * indicated P < 0.05 compared to Blank group, # indicated P < 0.05 compared to MDR group. Upper lane, A549 cells and lower lane, H1650 cells.

We used empty vector pCDNA with GFP to explore the efficiency of transfection. The ratio of plasmid/ Lipofectamine2000 was 1:2.5. The efficiency of the siRNA transfection into A549 cells and H1650 cells reached 80.2 % and 71.6 % respectively. To determine the change of MVIH expression after nedaplatin treatment, we performed a qRT-PCR in two lung cancer cell lines, A549 and H1650. MVIH siRNA and nedaplatin significantly reduced the MVIH expression in A549 and H1650 cells (**supplementary figure 1**). MTT assay was performed to examine cell proliferation after MHIV siRNA transfection. Results were shown in **Figure 1A**. The proliferation of cells in NDP treatment group and MVIH siRNA group was significantly lower than those in blank group, siRNA control group and MDR group, and the cell proliferation in MDR group was significantly higher than that in other groups (P <0.05) in both A549 and H1650 cells, suggesting MVIH promotes cell proliferation.

The colony formation results are shown in **Figure 1B**. Similar to the results of MTT, the number of colony formed in NDP group and MVIH siRNA group was significantly lower than that of the other three groups (P <0.05), and there was no significant difference between these two groups (P >0.05), which indicated that NDP and knockdown of MVIH might reduce cell colony formation. A549 and H1650 cells formed more colonies in other groups and there is no significance between blank group and siRNA control group (P >0.05).

### LncRNA MVIH and NDP treatment induced cell cycle arrest in lung cancer cells

**Figure 2:**
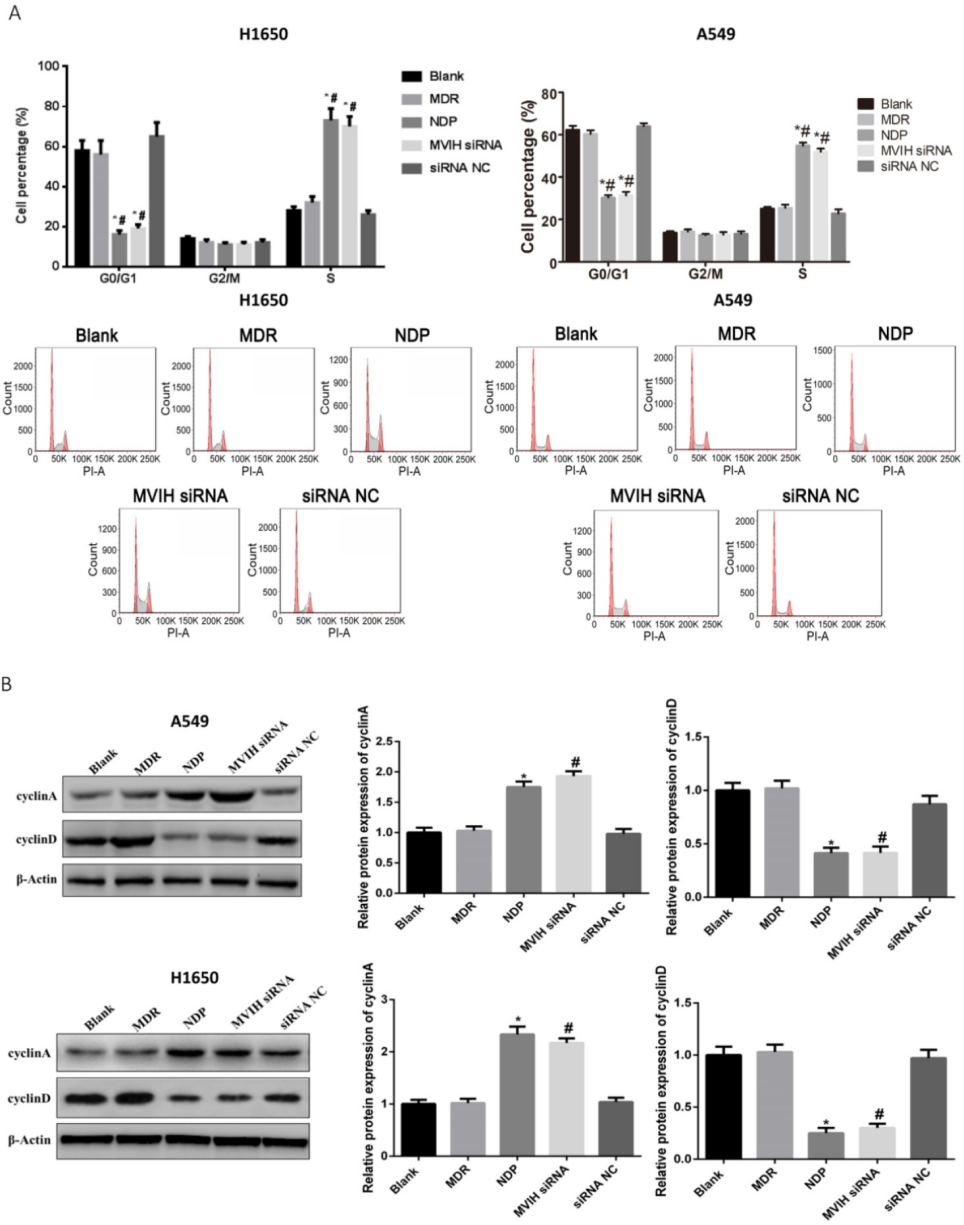
** Cell cycle change after nedaplatin treatment and knockdown of MVIH** A, cell cycle changes. A549, upper panel; H1650, lower panel. B, expression of cyclinA and cyclinD after nedaplatin treatment and knockdown of MVIH. A549, upper panel; H1650, lower panel. * indicated P < 0.05 compared to Blank group, # indicated P < 0.05 compared to MDR group.

Cell cycle was analyzed by flow cytometry. The results were shown in **Figure 2**. The percentage of cells in G2 / M phase in each group was not statistically different (P >0.05). NDP and MVIH siRNA significantly increased the proportion of cells in G0 / G1 phase and decrease the proportion of cells in S phase (P <0.05). There was no significant difference in cell cycle between blank group and MDR group (P >0.05). Results from Western blot showed decreased expression of cyclin A in cells of MVIH knock-down group. The expression of cyclin A in cells of NDP group was significantly higher than non-treatment group and MDR group (**Figure 2B**). These results suggested that NDP and knock-down of MVIH induced cell cycle arrest by increasing cyclin A.

### LncRNA MVIH siRNA and NDP treatment induced apoptosis in lung cancer cells

**Figure 3:**
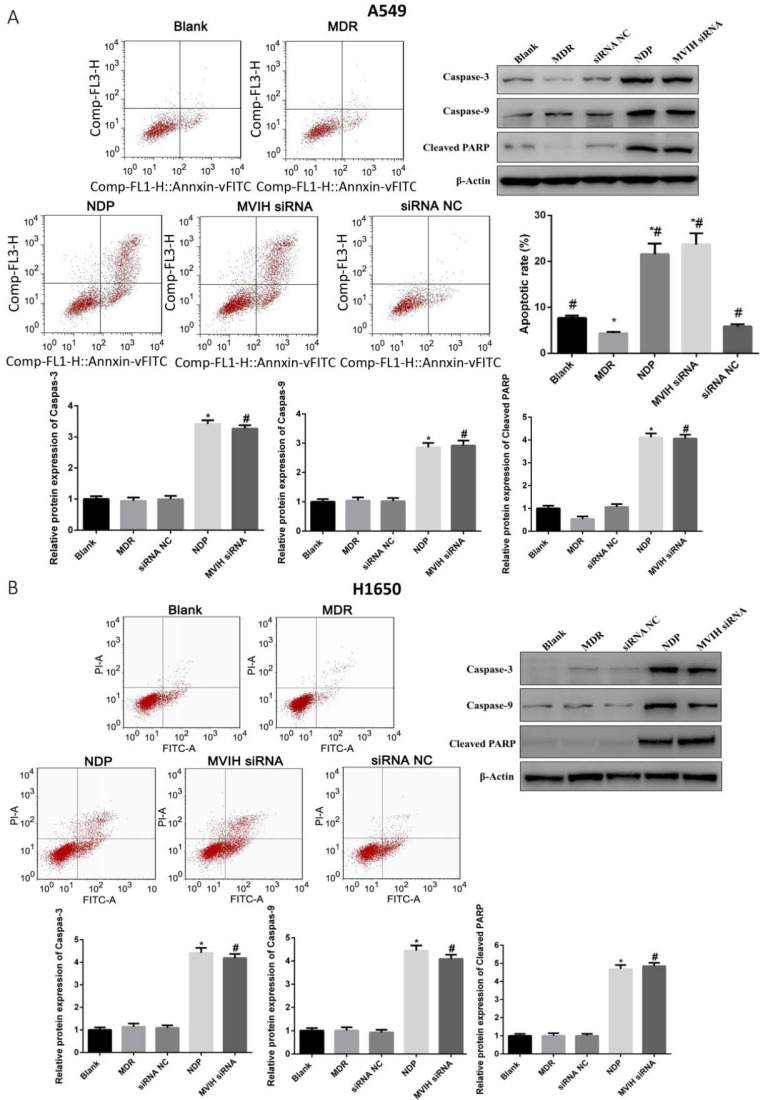
** Cell apoptosis after nedaplatin treatment and knockdown of MVIH**. A, A549 cells, B, H1650 cells. * indicated P < 0.05 compared to Blank group, # indicated P < 0.05 compared to MDR group.

As shown in **Figure 3**, the apoptotic rate of A549 and H1650 cells in MDR group was significantly lower than that in other groups (P <0.05). NDP and MVIH siRNA significantly promoted the apoptotic rate of A549 and H1650 cells. Although the apoptotic rate of MVIH siRNA group was higher than that of NDP group, there was no significant difference between the two groups (P >0.05). Apoptosis markers such as caspase-3, caspase-6 and cleaved PARP were significantly upregulated in MVIH knock-down and NDP group, compared to non-treatment group and MDR group **(Figure 3)**. These results demonstrated that knock-down of MVIH and NDP treatment induced cell apoptosis in lung cancer cells.

### LncRNA MVIH siRNA and NDP treatment reduced cell migration and invasion

**Figure 4:**
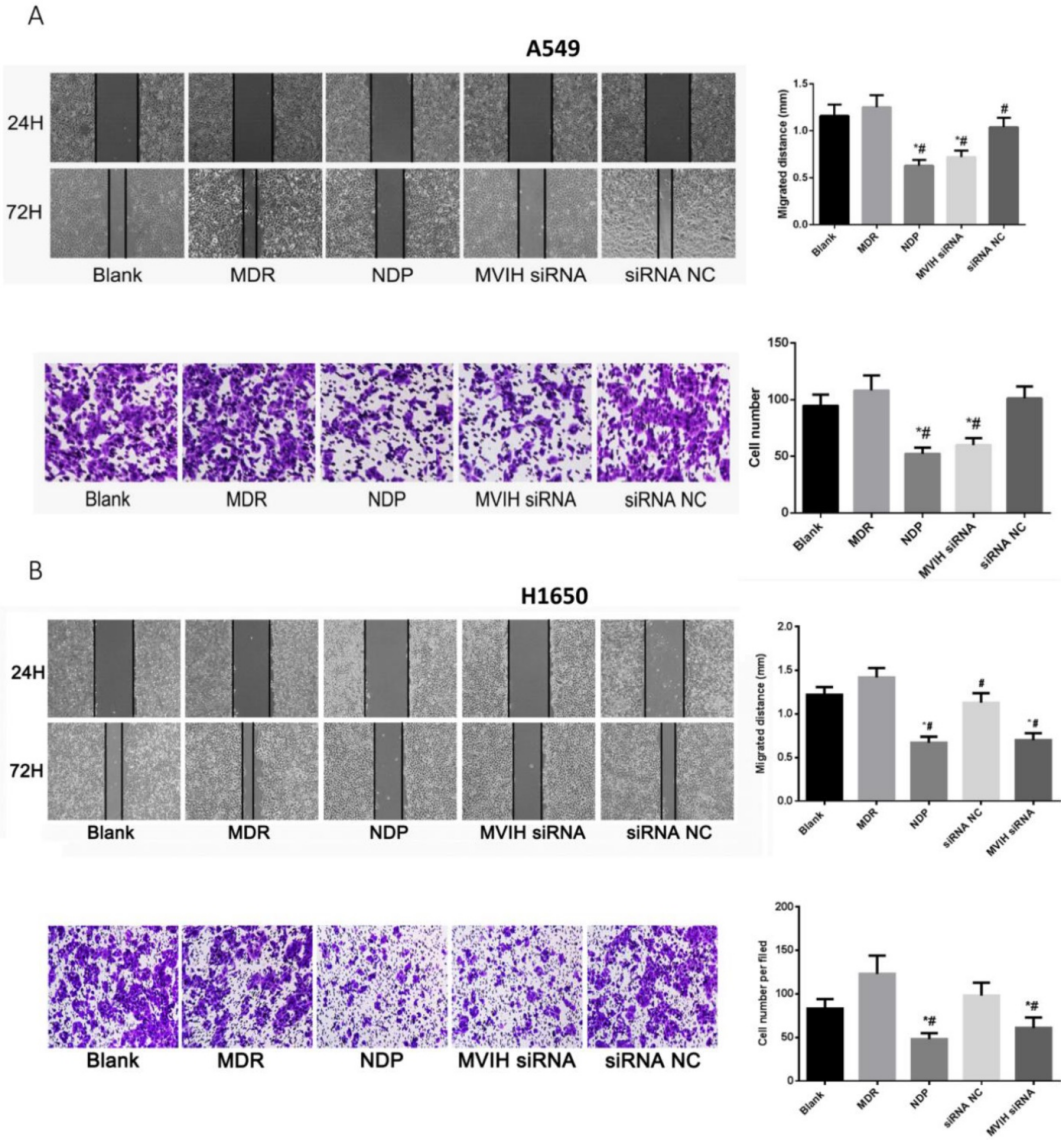
** Cell migration (A) and invasion (B) assay after nedaplatin treatment and knockdown of MVIH**. Upper panel, A549 cells and lower panel, H1650 cells. * indicated P < 0.05 compared to Blank group, # indicated P < 0.05 compared to MDR group.

As shown in **Figure 4A**, wound healing assay showed that cell migration ability of NDP and MVIH siRNA group was significantly lower than that of the other three groups (P <0.05). There was no significant difference in cell migration ability among blank group, MDR group and siRNA group. On the other hand, Transwell chamber assay (**Figure 4B**) showed that NDP and MVIH siRNA significantly reduce A549 and H1650 cells invasion, compared with the other three groups (P <0.05), suggesting that MVIH might promote cell invasion. There was no significant difference in invasive ability between blank group, MDR group and siRNA group. There was no significant difference between NPD and MVIH siRNA groups in terms of cell invasion (P >0.05).

### LncRNA MVIH siRNA and NDP treatment inhibited Epithelium Mesenchymal Transition (EMT)

**Figure 5:**
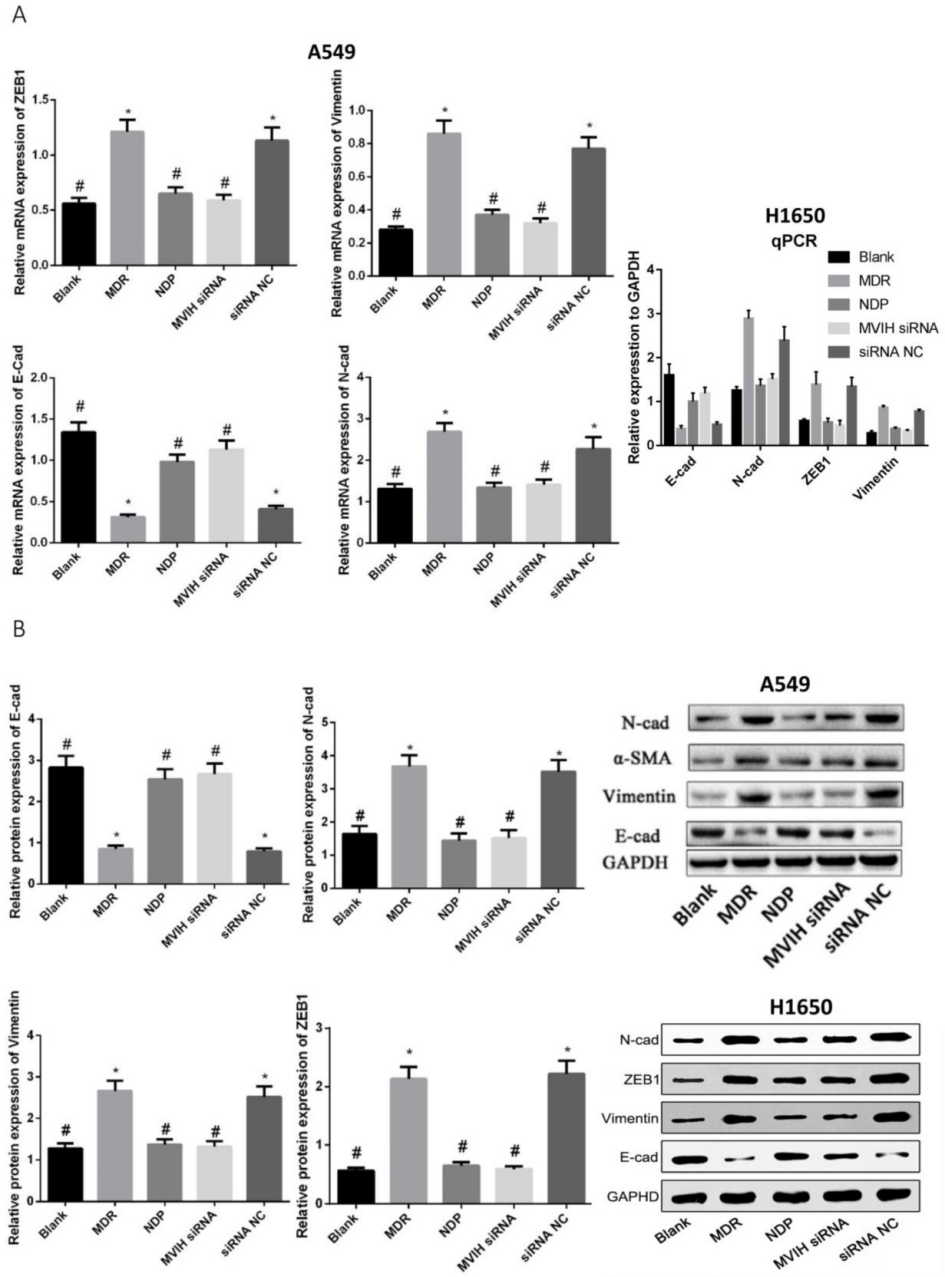
** Genes (A) and protein (B) expression after nedaplatin treatment and knockdown of MVIH.** E-cad, N-cad, ZEB1 and Vimentin mRNA and protein expression. * indicated P < 0.05 compared to Blank group, # indicated P < 0.05 compared to MDR group.

EMT markers were detected by qRT-PCR and Western Blot (**Figure 5**). The expression of E-cadherin (epithelium marker) in MDR group was significantly lower than that in Blank, NDP and MVIH siRNA group, while expression of N-cadherin, α-SMA and Vimentin in MDR group was significantly higher than that in Blank, NDP and MVIH siRNA groups (P <0.05), indicating MDR could induce EMT in A549 and H1650 cells, while MVIH siRNA and NDP treatment inhibited EMT.

### Knockdown of MVIH and NDP treatment reduced tumor growth and increased survival

**Figure 6:**
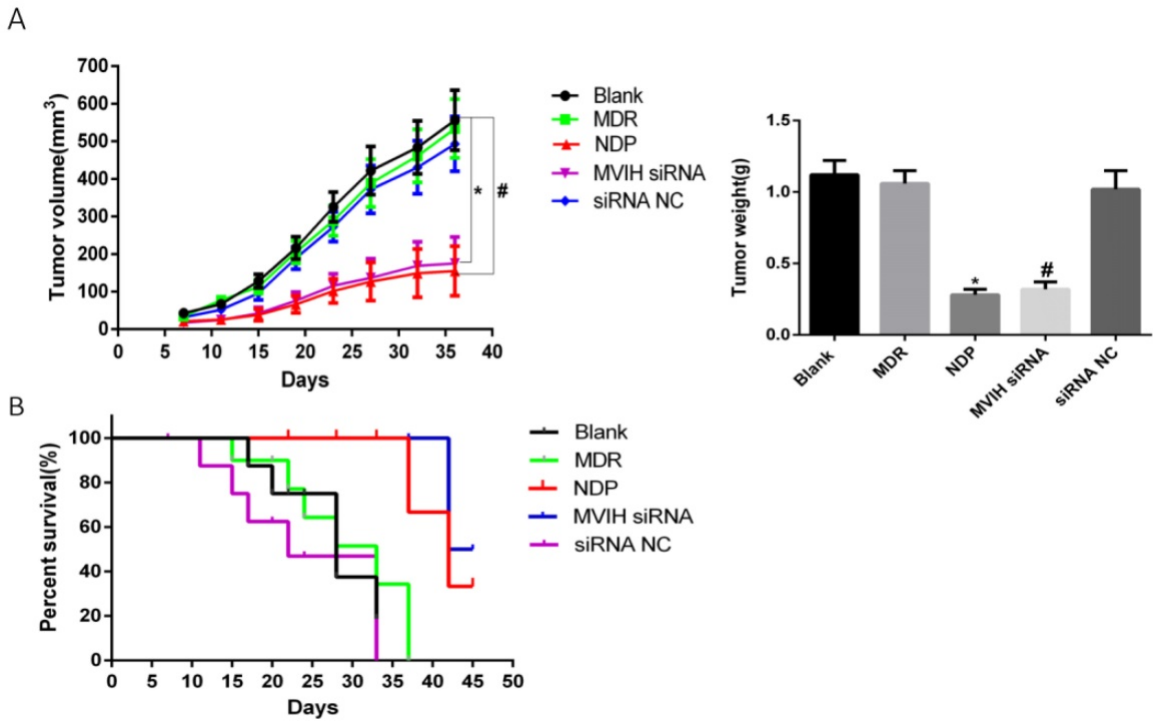
** Mouse tumor xenografts.** A, the growth curves and tumor weight over 36 days after lung cancer cells implantation. B, the overall survival curve of mice.

We established a mouse xenograft model to test the effect of MVIH and NDP treatment in lung cancer. Our results demonstrated a significant reduction of tumor size in MVIH siRNA and NDP group, comparing to blank, MDP and siRNA NC groups over all measuring time points (Figure 6A). This result suggested that knockdown of MVIH and NDP treatment effectively reduce tumor growth Moreover, survival analysis also suggested knockdown of MVIH and NDP treatment remarkedly prolong the overall survival of mice (Figure 6B).

## Discussion

NSCLC is the most common malignancy in the respiratory system, accounting for about 80% of all lung cancers. The current treatment for NSCLC includes surgery, radiotherapy and chemotherapy, with surgery being the most effective treatment 13. However, due to the fact that early diagnosis is unavailable, many patients were diagnosed as advanced lung cancer with metastasis, therefore missing the opportunity for surgical treatment. In this scenario, non-surgical treatment plays an important role in the comprehensive management of patients. Cisplatin is widely used in NSCLC chemotherapy, but sensitivity of cisplatin in NSCLC patients decreased after a period of treatment. The mechanisms by which tumor cells become less sensitive to cisplatin and develop drug resistance remain unclear. Any abnormality involving cell growth, cell cycle regulation, apoptosis, DNA damage repair and drug transport may cause loss of sensitivity to cisplatin 14-16.

Chemotherapy resistance can be categorized into primary and secondary drug resistance. Primary resistance refers to a subpopulation of tumor cells containing genes that are response for therapeutic resistance exists in initial tumor. It is characterized by tumor cells not response to chemotherapy, even if the concentration of drugs is sufficient. Secondary resistance (acquired resistance) refers to drug resistance appeared after chemotherapy, although patients show good curative effect in initial stage. Tumor gradually loses responses to chemotherapy, and tumor volume increases again 17. Cisplatin is widely used in lung cancer chemotherapy, and cisplatin resistance has become a problem in clinics. Therefore, development of chemotherapeutic drugs that are effective for tumors with cisplatin resistance (primary/secondary) is important for cancer treatments.

Platinum-based drugs, particularly cisplatin and carboplatin are widely used as antineoplastic agents. Due to toxicity issues, other platinum derivatives have been developed. Nedaplatin is one example of such new drugs. Nedaplatin consists of two ammine ligands and a dianion derived from glycolic acid. A phase II clinical study in Japan showed that NDP is very effective in the treatment of small cell lung cancer and non-small cell lung cancer 11. More importantly, it was reported that only incomplete cross-resistance with cisplatin and carboplatin was observed in patients 18, 19.

LncRNA MVIH was initially discovered in hepatocellular carcinoma, and abnormal MVIH expression in various cancer cells was then detected. Studies have shown that the expression of MVIH in NSCLC tissues was significantly increased 10, and *in vitro* experiments confirmed that the construction and transfection of specific siRNA to interference MVIH expression significantly inhibit the proliferation and invasion of osteosarcoma cells 20.

Our results suggested that MVIH is up-regulated in nedaplatin treated cells, which suggests MVIH involve in drug resistance in NSCLC after cisplatin treatment. NDP treatment attenuates the expression of MVIH in A549 and H1650 cells. Our results also revealed that NDP had a significant inhibitory effect on cisplatin secondary drug-resistance and it had incomplete cross-resistance with cisplatin. Its mechanism of action may be achieved by down-regulating MVIH expression. Colony formation assay also demonstrated that MVIH promotes tumor cell colony formation. Moreover, our results showed that NPD could reduce migration and invasion ability of A549 and H1650 cells, which is most likely caused by downregulating MVIH expressions.

Our results from flow cytometry indicated that NDP block A549 and H1650 cell cycle in S phase. This result is consistent with one previous study, which used NDP on human gastric cancer AZ-521 cells 21. Other studies also found that different cisplatin drugs have similar effects on cell cycle in different cancer types 22-24, suggesting that cell cycle blockage in S phase may be one of the mechanisms in which NDP-induced apoptosis 25. In our study, we found that apoptotic rate increased significantly in NDP or MVIH siRNA treated MDR cells, indicating that NDP could improve chemotherapeutic sensitivity of A549 and H1650 cells and promote apoptosis by down-regulating MVIH.

The epithelial-mesenchymal transition (EMT) is a process by which epithelial cells lose their cell polarity and cell-cell adhesion, to become mesenchymal cells and gain migratory and invasive properties. EMT is essential for numerous developmental processes including neural tube formation. EMT has also been shown in the initiation of metastasis for cancer progression. Zeb1 is an important transcription factor in the process of EMT and initiates EMT by binding to E-BOX in the epithelial cell marker E-cadherin gene transcription promoter sequence 26, 27. Zeb1 has been recognized as essential for the maintenance of cell properties. Normal epithelial tissue and well-differentiated cancers do not express or rarely express this factor. On the contrast, ZEB1 gene is often overexpressed in poorly differentiated malignant cells, especially at early age of invasion 28. Meanwhile, studies have shown that, ZEB1 exerted a direct inhibitory effect on E-cadherin gene through E-Box regulation. The complexes formed by ZEB1 and histone deacetylase may be involved in transcriptional cleavage of E-cadherin and its expression was inhibited. In this study, we found that the expression of stromal cell markers such as ZEB1, Vimentin and N-cadherin in MDR cells was significantly higher than that in untreated A549 and H1650 cells. Moreover, the expression of E-cadherin was significantly decreased, indicating that EMT process may be involved in NSCLC drug resistance. After treatment with NDP or MVIH siRNA, the expression of stromal cell markers ZEB1, Vimentin and N-cadherin in A549 and H1650 cells were significantly down-regulated, while the expression of E-cadherin was significantly up-regulated, suggesting that NDP can reverse EMT process. This result may be caused by reducing the expression of MVIH.

In summary, NSCLC A549 and H1650 cells were used to examine the effect of nedaplatin on chemotherapy. Nedaplatin decreases the expression of lncRNA MVIH and reverses the EMT process, thereby restoring drug sensitivity of non-small cell lung cancer cells. This study provided a rational for using nedaplatin for the treatment of NSCLC.

## Supplementary Material

Supplementary figures and tables.

## Figures and Tables

**Table 1:** Primers

Gene	Sequence Forward	Sequence reverse
Zeb1	5'-TAC AGA ACC CAA CTT GAA CGT CAC A-3'	5'-GAT TAC ACC CAG ACT GCG TCA CA-3'
vimentin	5′-GAGAACTTTGCCGTTGAAGC-3′	5′-GCTTCCTGTAGGTGGCAATC-3′
N-cadherin	5′-ACAGTGGCCACCTACAAAGG-3′	5′-CCGAGATGGGGTTGATAATGN-3′
E-cadherin	5′-TGCCCAGAAAATGAAAAAGG-3′	5′-GTGTATGTGGCAATGCGTTC-3′
