# Supplementary material for: Nedaplatin reduces multidrug resistance of non-small cell lung cancer by downregulating the expression of long non-coding RNA MVIH
Source: J Cancer. 2020 Jan 1;11(3):559–69. doi: 10.7150/jca.35792 (PMC6959054; doi:10.7150/jca.35792)

Supplementary Figure 1. Bar plot of MVIH expression after siRNA transfection. The expression of MVIH was relative to internal reference gene. *, p<0.05.

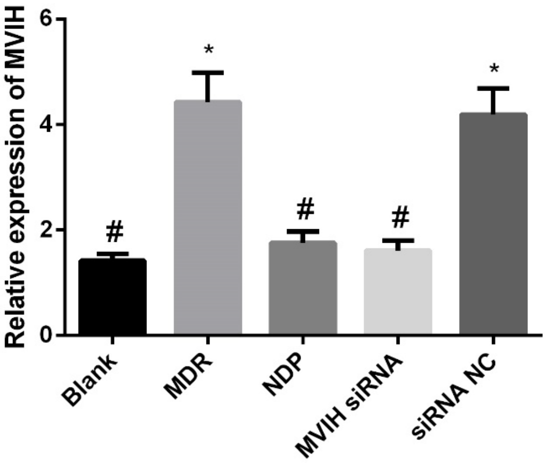

Supplement: Supplementary file 1 — Supplementary figures and tables. [file jcav11p0559s1.pdf]
